# On the space of Laplace transformable distributions

**DOI:** 10.1007/s13398-020-00907-2

**Published:** 2020-08-12

**Authors:** Andreas Debrouwere, Eduard A. Nigsch

**Affiliations:** 1 Department of Mathematics: Analysis, Logic and Discrete Mathematics, Ghent University, Krijgslaan 281, 9000 Gent, Belgium; 2 Institute for Analysis and Scientific Computing, TU Vienna, Wiedner Hauptstraße 8–10, 1040 Vienna, Austria

**Keywords:** Laplace transform, Distributions, Ultrabornological (PLS)-spaces, Short-time Fourier transform, Primary 46F05, 46A13, Secondary 81S30

## Abstract

We show that the space ${\mathcal {S}}'(\Gamma )$ of Laplace transformable distributions, where $\Gamma \subseteq {\mathbb {R}}^d$ is a non-empty convex open set, is an ultrabornological (PLS)-space. Moreover, we determine an explicit topological predual of ${\mathcal {S}}'(\Gamma )$.

## Introduction

Schwartz introduced the space ${\mathcal {S}}'(\Gamma )$ of Laplace transformable distributions as

$$\begin{aligned} {\mathcal {S}}'(\Gamma ) = \{ f \in {\mathcal {D}}'({\mathbb {R}}^d)\ |\ e^{-\xi \cdot x} f(x) \in {\mathcal {S}}'({\mathbb {R}}^d_x)\ \forall \xi \in \Gamma \}, \end{aligned}$$

where $\Gamma \subseteq {\mathbb {R}}^d$ is a non-empty convex set [1, p. 303]. This space is endowed with the projective limit topology with respect to the mappings ${\mathcal {S}}'(\Gamma ) \rightarrow {\mathcal {S}}'({\mathbb {R}}^d)$, $f \mapsto e^{-\xi \cdot x} f(x)$ for $\xi \in \Gamma$. The second author together with Kunzinger and Ortner [2] recently presented two new proofs of Schwartz’s exchange theorem for the Laplace transform of vector-valued distributions [3, Prop. 4.3, p. 186]. Their methods required them to show that ${\mathcal {S}}'(\Gamma )$ is complete, nuclear and dual-nuclear [2, Lemma 5]. Following a suggestion of Ortner, in this article, we further study the locally convex structure of the space ${\mathcal {S}}'(\Gamma )$.

In order to be able to apply functional analytic tools such as De Wilde’s open mapping and closed graph theorems [4, Theorem 24.30 and Theorem 24.31] or the theory of the derived projective limit functor [5], it is important to determine when a space is ultrabornological. This is usually straightforward if the space is given by a suitable inductive limit; in fact, ultrabornological spaces are exactly the inductive limits of Banach spaces [4, Proposition 24.14]. The situation for projective limits, however, is more complicated. Particularly, this applies to the class of (PLS)-spaces (i.e., countable projective limits of (DFS)-spaces). The problem of ultrabornologicity has been extensively studied in this class, both from an abstract point of view as for concrete function and distribution spaces; see the survey article [6] of Domański and the references therein.

In the last part of his doctoral thesis [7, Chap. II, Thm. 16, p. 131], Grothendieck showed that the convolutor space ${\mathcal {O}}_C'$ is ultrabornological. He proved that ${\mathcal {O}}_C'$ is isomorphic to a complemented subspace of the sequence space $s {\widehat{\otimes }} s'$ and verified directly that the latter space is ultrabornological. Much later, a different proof was given by Larcher and Wengenroth using homological methods [8]. The first author and Vindas [9] extended this result to a considerably wider setting by studying the locally convex structure of a general class of weighted convolutor spaces. More precisely, they characterized when such spaces are ultrabornological and determined explicit topological preduals for them. One of their main tools is a topological description of these convolutor spaces in terms of the short-time Fourier transform (STFT).

In this work, we will identify ${\mathcal {S}}'(\Gamma )$ with a particular instance of the convolutor spaces considered in [9]. To this end, we make a detailed study of the mapping properties of the STFT on ${\mathcal {S}}'(\Gamma )$. Once this identification has been established, we use Theorem 1.1 from [9] (see also Theorem 4.2 below) to show that ${\mathcal {S}}'(\Gamma )$ is an ultrabornological (PLS)-space and that it admits a weighted (LF)-space of smooth functions on ${\mathbb {R}}^d$ as a topological predual.

## Weighted spaces of continuous functions

For formulating the mapping properties of the STFT we recall the following notions from [9, 10].

Each non-negative function *v* on ${\mathbb {R}}^d$ defines a weighted seminorm on $C({\mathbb {R}}^d)$ by

$$\begin{aligned} \Vert f\Vert _{v} \mathrel {\mathop :}=\sup _{x \in {\mathbb {R}}^d} \left| f(x)\right| v(x). \end{aligned}$$

We endow the space

$$\begin{aligned} Cv({\mathbb {R}}^d) \mathrel {\mathop :}=\{ f \in C({\mathbb {R}}^d)\ |\ \Vert f\Vert _{v} < \infty \} \end{aligned}$$

with this seminorm; it is a Banach space if *v* is positive and continuous. A pointwise decreasing sequence ${\mathcal {V}} = (v_N)_{N \in {\mathbb {N}}}$ of positive continuous functions on ${\mathbb {R}}^d$ is called a *decreasing weight system*. With this, we define the (*LB*)-space

$$\begin{aligned} {\mathcal {V}}C({\mathbb {R}}^d)\mathrel {\mathop :}=\varinjlim _{N \in {\mathbb {N}}} Cv_N({\mathbb {R}}^d). \end{aligned}$$

We consider the following condition on a decreasing weight system ${\mathcal {V}}$, see [10, p. 114]:

V $$\begin{aligned} \forall N \in {\mathbb {N}}\, \exists M > N \, : \, \lim _{\left| x\right| \rightarrow \infty }\frac{v_M(x)}{v_N(x)} = 0. \end{aligned}$$

The *maximal Nachbin family associated with* ${\mathcal {V}}$ is defined to be the family ${\overline{V}}={\overline{V}}({\mathcal {V}})$ consisting of all non-negative upper semicontinuous functions *v* on ${\mathbb {R}}^d$ such that

$$\begin{aligned} \forall N \in {\mathbb {N}}\, : \,\sup _{x \in {\mathbb {R}}^d} \frac{v(x)}{v_N(x)} < \infty . \end{aligned}$$

The *projective hull of* ${\mathcal {V}}C({\mathbb {R}}^d)$ is defined as

$$\begin{aligned} C{\overline{V}}({\mathbb {R}}^d) \mathrel {\mathop :}=\{ f \in C({\mathbb {R}}^d)\ |\ \Vert f\Vert _{v} < \infty \ \forall v \in {\overline{V}} \}. \end{aligned}$$

and endowed with the locally convex topology generated by the system of seminorms $\{ \Vert \,\cdot \,\Vert _{v} \, | \, v \in {\overline{V}} \}$. The spaces ${\mathcal {V}}C({\mathbb {R}}^d)$ and $C{\overline{V}}({\mathbb {R}}^d)$ always coincide as sets and, if ${\mathcal {V}}$ satisfies condition (V), also as locally convex spaces [10, Thm. 1.3 (d), p. 118].

A pointwise increasing sequence ${\mathcal {W}} = (w_N)_{N \in {\mathbb {N}}}$ of positive continuous functions on ${\mathbb {R}}^d$ is called an *increasing weight system*. Given such a system, we define the Fréchet space

$$\begin{aligned} {\mathcal {W}}C({\mathbb {R}}^d)\mathrel {\mathop :}=\varprojlim _{N \in {\mathbb {N}}} Cw_N({\mathbb {R}}^d). \end{aligned}$$

We consider the following conditions on an increasing weight system ${\mathcal {W}}$:

2.1 $$\begin{aligned}&\forall N \in {\mathbb {N}}\, \exists M > N \, : \, \lim _{\left| x\right| \rightarrow \infty }\frac{w_N(x)}{w_M(x)} = 0, \end{aligned}$$

2.2 $$\begin{aligned}&\forall N \in {\mathbb {N}}\, \exists M > N : \frac{w_N}{w_M} \in L^1({\mathbb {R}}^d), \end{aligned}$$

2.3 $$\begin{aligned}&\forall N \in {\mathbb {N}}\, \exists M_1,M_2 \ge N \, \exists C > 0 \, \forall x,y \in {\mathbb {R}}^d : w_N(x+y) \le C w_{M_1}(x) w_{M_2}(y). \end{aligned}$$

In the next lemma, we obtain a concrete representation of the $\varepsilon$-tensor product of weighted spaces of continuous functions.

### Lemma 2.1

Let ${\mathcal {W}} = (w_N)_{N \in {\mathbb {N}}}$ be an increasing weight system and ${\mathcal {V}} = (v_n)_{n \in {\mathbb {N}}}$ a decreasing weight system satisfying (*V*). Then, we have the identification

$$\begin{aligned} {\mathcal {W}}C({\mathbb {R}}^d_x) {\widehat{\otimes }}_\varepsilon {\mathcal {V}}C({\mathbb {R}}^d_\xi ) = \{ f \in C({\mathbb {R}}^{2d}_{x,\xi })\ |\ \forall N \in {\mathbb {N}}\ \exists n \in {\mathbb {N}}: \Vert f\Vert _{w_N \otimes v_n} < \infty \}, \end{aligned}$$

where we set $\Vert f\Vert _{w \otimes v} \mathrel {\mathop :}=\sup _{(x,\xi ) \in {\mathbb {R}}^{2d}} \left| f(x,\xi )\right| w(x) v(\xi )$ for non-negative functions *w*, *v* on ${\mathbb {R}}^d$. Moreover, $f \in C({\mathbb {R}}^{2d}_{x,\xi })$ belongs to ${\mathcal {W}}C({\mathbb {R}}^d_x) {\widehat{\otimes }}_\varepsilon {\mathcal {V}}C({\mathbb {R}}^d_\xi )$ if and only if $\Vert f\Vert _{w_N \otimes v} < \infty$ for all $N \in {\mathbb {N}}$ and $v \in {\overline{V}}$. Consequently, the topology of ${\mathcal {W}}C({\mathbb {R}}^d_x) {\widehat{\otimes }}_\varepsilon {\mathcal {V}}C({\mathbb {R}}^d_\xi )$ is generated by the system of seminorms $\{ \Vert \, \cdot \, \Vert _{w_N \otimes v} \, | \, N \in {\mathbb {N}}, v \in {\overline{V}} \}$.

### Proof

This follows from the fact that the $\varepsilon$-tensor product commutes with projective limits and [10, Thm. 3.1 (c), p. 137]. $\square$

## The short-time Fourier transform on ${\mathcal {D}}'({\mathbb {R}}^d)$

The translation and modulation operators are denoted by $T_xf(t) = f(t-x)$ and $M_\xi f(t) = e^{2\pi i \xi \cdot t} f(t)$ for $x, \xi \in {\mathbb {R}}^d$. The *short-time Fourier transform (STFT)* of a function $f \in L^2({\mathbb {R}}^d)$ with respect to a window function $\psi \in L^2({\mathbb {R}}^d)$ is defined as

$$\begin{aligned} V_\psi f(x,\xi ) \mathrel {\mathop :}=(f, M_\xi T_x\psi )_{L^2} = \int _{{\mathbb {R}}^d} f(t) \overline{\psi (t-x)}e^{-2\pi i \xi \cdot t}\, \mathrm{d}t , \qquad (x, \xi ) \in {\mathbb {R}}^{2d}, \end{aligned}$$

where $(\cdot ,\cdot )_{L^2}$ denotes the inner product on $L^2({\mathbb {R}}^d)$. We have that $\Vert V_\psi f\Vert _{L^2({\mathbb {R}}^{2d})} = \Vert \psi \Vert _{L^2}\Vert f\Vert _{L^2}$. In particular, the mapping $V_\psi :L^2({\mathbb {R}}^d) \rightarrow L^2({\mathbb {R}}^{2d})$ is continuous. The adjoint of $V_\psi$ is given by the weak integral

$$\begin{aligned} V^*_\psi F = \int \int _{{\mathbb {R}}^{2d}} F(x,\xi ) M_\xi T_x\psi \, \mathrm{d}x \, \mathrm{d}\xi , \qquad F \in L^2({\mathbb {R}}^{2d}). \end{aligned}$$

If $\psi \ne 0$ and $\gamma \in L^2({\mathbb {R}}^d)$ is a synthesis window for $\psi$, that is, $(\gamma , \psi )_{L^2} \ne 0$, then

$$\begin{aligned} \frac{1}{(\gamma , \psi )_{L^2}} V^*_\gamma \circ V_\psi = {{\,\mathrm{id}\,}}_{L^2({\mathbb {R}}^d)}. \end{aligned}$$

We refer to [11] for further properties of the STFT.

Next, we explain how the STFT can be extended to the space of distributions; see [9, Sect. 2] for details and proofs. We set ${\mathcal {V}}_{{\text {pol}}} = ((1+ \left| \, \cdot \, \right| )^{-N})_{N \in {\mathbb {N}}}.$ Fix a window function $\psi \in {\mathcal {D}}({\mathbb {R}}^d)$. For $f \in {\mathcal {D}}'({\mathbb {R}}^d)$ we define

$$\begin{aligned} V_\psi f(x,\xi ) \mathrel {\mathop :}=\langle f, \overline{M_\xi T_x\psi } \rangle , \qquad (x,\xi ) \in {\mathbb {R}}^{2d}. \end{aligned}$$

Clearly, $V_\psi f$ is a continuous function on ${\mathbb {R}}^{2d}$. In fact,

$$\begin{aligned} V_\psi :{\mathcal {D}}'({\mathbb {R}}^d) \rightarrow C({\mathbb {R}}^d_x) {\widehat{\otimes }}_\varepsilon {\mathcal {V}}_{{\text {pol}}}C({\mathbb {R}}^d_\xi ) \end{aligned}$$

is a well-defined continuous mapping [9, Lemma 2.2]. We *define* the adjoint STFT of an element $F \in C({\mathbb {R}}^d_x) {\widehat{\otimes }}_\varepsilon {\mathcal {V}}_{{\text {pol}}}C({\mathbb {R}}^d_\xi )$ as the distribution

$$\begin{aligned} \langle V^*_\psi F, \varphi \rangle \mathrel {\mathop :}=\int \int _{{\mathbb {R}}^{2d}} F(x,\xi ) V_{{\overline{\psi }}}\varphi (x, -\xi )\,\mathrm{d}x \,\mathrm{d}\xi , \qquad \varphi \in {\mathcal {D}}({\mathbb {R}}^d). \end{aligned}$$

Then,

$$\begin{aligned} V^*_\psi :C({\mathbb {R}}^d_x) {\widehat{\otimes }}_\varepsilon {\mathcal {V}}_{{\text {pol}}}C({\mathbb {R}}^d_\xi ) \rightarrow {\mathcal {D}}'({\mathbb {R}}^d) \end{aligned}$$

is a well-defined continuous mapping by [9, Prop. 2.2]. Finally, if $\psi \ne 0$ and $\gamma \in {\mathcal {D}}({\mathbb {R}}^d)$ is a synthesis window for $\psi$, then the following reconstruction formula holds [9, Prop. 2.4]:

3.1 $$\begin{aligned} \frac{1}{(\gamma , \psi )_{L^2}} V^*_\gamma \circ V_\psi = {\text {id}}_{{\mathcal {D}}'({\mathbb {R}}^d)}. \end{aligned}$$

## Duals of inductive limits of weighted spaces of smooth functions

Let *v* be a non-negative function on ${\mathbb {R}}^d$ and $n \in {\mathbb {N}}$. We define ${\mathcal {B}}^n_v({\mathbb {R}}^d)$ as the seminormed space consisting of all $\varphi \in C^n({\mathbb {R}}^d)$ such that

$$\begin{aligned} \Vert \varphi \Vert _{v,n} \mathrel {\mathop :}=\max _{\left| \alpha \right| \le n} \sup _{x \in {\mathbb {R}}^d} \left| \partial ^{\alpha }\varphi (x)\right| v(x) < \infty . \end{aligned}$$

As before, ${\mathcal {B}}^n_v({\mathbb {R}}^d)$ is a Banach space if *v* is positive and continuous. Let ${\mathcal {W}} = (w_N)_{N \in {\mathbb {N}}}$ be an increasing weight system. We define the (LF)-space

$$\begin{aligned} {\mathcal {B}}_{{\mathcal {W}}^\circ }({\mathbb {R}}^d) := \varinjlim _{N \in {\mathbb {N}}} \varprojlim _{n \in {\mathbb {N}}} {\mathcal {B}}^n_{1/w_N}({\mathbb {R}}^d). \end{aligned}$$

We endow the dual space ${\mathcal {B}}'_{{\mathcal {W}}}({\mathbb {R}}^d) \mathrel {\mathop :}=({\mathcal {B}}_{{\mathcal {W}}^\circ }({\mathbb {R}}^d))'$ with the strong topology. If ${\mathcal {W}}$ satisfies (2.1), then ${\mathcal {D}}({\mathbb {R}})$ is densely and continuously included in ${\mathcal {B}}_{{\mathcal {W}}^\circ }({\mathbb {R}}^d)$ and therefore ${\mathcal {B}}'_{{\mathcal {W}}}({\mathbb {R}}^d)$ is a vector subspace of ${\mathcal {D}}'({\mathbb {R}}^d)$.

On the other hand, we define the convolutor space

$$\begin{aligned} {\mathcal {O}}'_{C,{\mathcal {W}}}({\mathbb {R}}^d) \mathrel {\mathop :}=\{ f \in {\mathcal {D}}'({\mathbb {R}}^d)\ |\ f *\varphi \in {\mathcal {W}}C({\mathbb {R}}^d)\ \forall \varphi \in {\mathcal {D}}({\mathbb {R}}^d) \}. \end{aligned}$$

For $f \in {\mathcal {O}}'_{C,{\mathcal {W}}}({\mathbb {R}}^d)$ fixed, the mapping

$$\begin{aligned} {\mathcal {D}}({\mathbb {R}}^d) \rightarrow {{\mathcal {W}}}C({\mathbb {R}}^d),\quad \varphi \mapsto f *\varphi \end{aligned}$$

is continuous, as follows from the closed graph theorem. We endow ${\mathcal {O}}'_{C,{\mathcal {W}}}({\mathbb {R}}^d)$ with the topology induced via the embedding

$$\begin{aligned} {\mathcal {O}}'_{C,{\mathcal {W}}}({\mathbb {R}}^d) \rightarrow L_\beta ({\mathcal {D}}({\mathbb {R}}^d), {\mathcal {W}}C({\mathbb {R}}^d)),\quad f \mapsto [\varphi \mapsto f *\varphi ], \end{aligned}$$

where $\beta$ denotes the topology of uniform convergence on bounded sets.

In [9] the structural and topological properties of the spaces ${\mathcal {B}}'_{{\mathcal {W}}}({\mathbb {R}}^d)$ and ${\mathcal {O}}'_{C,{\mathcal {W}}}({\mathbb {R}}^d)$ are discussed. We now present the main results of this paper and refer to [9] for more details and proofs.^1^

### Proposition 4.1

[9, Prop. 4.2] Let ${\mathcal {W}}$ be an increasing weight system satisfying (2.1), (2.2) and (2.3) and let $\psi \in {\mathcal {D}}({\mathbb {R}}^d)$. Then, the mappings

$$\begin{aligned} V_\psi :{\mathcal {O}}'_{C,{\mathcal {W}}}({\mathbb {R}}^d) \rightarrow {\mathcal {W}}C({\mathbb {R}}^d_x) {\widehat{\otimes }}_\varepsilon {\mathcal {V}}_{{\text {pol}}}C({\mathbb {R}}^d_\xi ) \end{aligned}$$

and

$$\begin{aligned} V^*_\psi :{\mathcal {W}}C({\mathbb {R}}^d_x) {\widehat{\otimes }}_\varepsilon {\mathcal {V}}_{{\text {pol}}}C({\mathbb {R}}^d_\xi ) \rightarrow {\mathcal {O}}'_{C,{\mathcal {W}}}({\mathbb {R}}^d) \end{aligned}$$

are well-defined and continuous.

### Theorem 4.2

[9, Thm. 3.4, Thm. 4.6 and Thm. 4.15] Let ${\mathcal {W}} = (w_N)_{N \in {\mathbb {N}}}$ be an increasing weight system satisfying (2.1), (2.2) and (2.3). Then, ${\mathcal {B}}'_{{\mathcal {W}}}({\mathbb {R}}^d) = {\mathcal {O}}'_{C,{\mathcal {W}}}({\mathbb {R}}^d)$ as sets and the inclusion mapping ${\mathcal {B}}'_{{\mathcal {W}}}({\mathbb {R}}^d) \rightarrow {\mathcal {O}}'_{C,{\mathcal {W}}}({\mathbb {R}}^d)$ is continuous. Moreover, the following statements are equivalent:

(i): ${\mathcal {B}}'_{{\mathcal {W}}}({\mathbb {R}}^d) = {\mathcal {O}}'_{C,{\mathcal {W}}}({\mathbb {R}}^d)$ as locally convex spaces.
(ii): ${\mathcal {O}}'_{C,{\mathcal {W}}}({\mathbb {R}}^d)$ is an ultrabornological (PLS)-space.
(iii): The (LF)-space ${\mathcal {B}}_{{\mathcal {W}}^\circ }({\mathbb {R}}^d)$ is complete.
(iv): $${\mathcal {W}}$$ satisfies
  4.1 $$\begin{aligned}&\forall N \in {\mathbb {N}}\, \exists M \ge N \, \forall P \ge M \, \exists \theta \in (0,1) \, \exists C > 0 \, \forall x \in {\mathbb {R}}^d: \nonumber \\&{w_N(x)}^{1-\theta }{w_P(x)}^{\theta } \le Cw_M(x). \end{aligned}$$

### Remark 4.3

Condition (4.1) is closely connected with D. Vogt’s condition $(\Omega )$ that plays an essential role in the structure and splitting theory for Fréchet spaces.

## The space ${\mathcal {S}}'(\Gamma )$

Our next goal is to characterize ${\mathcal {S}}'(\Gamma )$ in terms of the STFT.

Let $\emptyset \ne \Gamma \subseteq {\mathbb {R}}^d$ be open and convex. We denote by ${\text {CCS}}(\Gamma )$ the family of all non-empty compact convex subsets of $\Gamma$ and by ${\mathfrak {B}}({\mathcal {S}}({\mathbb {R}}^d))$ the family of all bounded subsets of ${\mathcal {S}}({\mathbb {R}}^d$). The topology of ${\mathcal {S}}'(\Gamma )$ can easily be described by a system of concrete seminorms which essentially is due to Schwartz [1, p. 301]; for this, note that the system of convex hulls of finite sets is cofinal in ${\text {CCS}}(\Gamma )$:

### Lemma 5.1

[1, p. 301] Let $\emptyset \ne \Gamma \subseteq {\mathbb {R}}^d$ be open and convex. For all $K \in {\text {CCS}}(\Gamma )$ and $B \in {\mathfrak {B}}({\mathcal {S}}({\mathbb {R}}^d))$ we have that

$$\begin{aligned} p_{K,B}(f) \mathrel {\mathop :}=\sup _{\eta \in K} \sup _{\varphi \in B} \left| \langle e^{-\eta \cdot x}f(x), \varphi (x) \rangle \right| < \infty , \qquad f \in {\mathcal {S}}'(\Gamma ). \end{aligned}$$

Moreover, the topology of ${\mathcal {S}}'(\Gamma )$ is generated by the system of seminorms $\{p_{K,B} \, | \, K \in {\text {CCS}}(\Gamma ), B \in {\mathfrak {B}}({\mathcal {S}}({\mathbb {R}}^d))\}$.

We need to introduce some additional terminology. Given a non-empty compact convex subset *K* of ${\mathbb {R}}^d$, we define its *supporting function* as

$$\begin{aligned} h_K(x) =\max _{\eta \in K} x \cdot \eta , \qquad x \in {\mathbb {R}}^d. \end{aligned}$$

It is clear from the definition that $h_K$ is subadditive and positive homogeneous of degree one. In particular, $h_K$ is convex. Supporting functions have the following elementary properties.

### Lemma 5.2

[12, Cor. 1.8.2 and Prop. 1.8.3] Let $K_1$ and $K_2$ be non-empty compact convex subsets of ${\mathbb {R}}^d$.

(a): $K_1 \subseteq K_2$ if and only if $h_{K_1}(x) \le h_{K_2}(x)$ for all $x \in {\mathbb {R}}^d$.
(b): $$h_{K_1+K_2}(x) = h_{K_1}(x) + h_{K_2}(x)$$ for all $$x \in {\mathbb {R}}^d$$.

### Example 5.3

For $r > 0$ we have $h_{{\overline{B}}(0,r)}(x) = r \left| x\right|$ for all $x \in {\mathbb {R}}^d$, where ${\overline{B}}(0,r)$ denotes the closed ball in ${\mathbb {R}}^d$ centered at the origin with radius *r*. Next, let *K* be a non-empty compact convex subset of ${\mathbb {R}}^d$ and $\varepsilon > 0$. We set $K_\varepsilon = K + {\overline{B}}(0,\varepsilon )$. Lemma 5.2 and the above yield that $h_{K_\varepsilon }(x) = h_K(x) + \varepsilon \left| x\right|$ for all $x \in {\mathbb {R}}^d$.

Let $\emptyset \ne \Gamma \subseteq {\mathbb {R}}^d$ be open and convex and let $(K_N)_{N \in {\mathbb {N}}} \subset {\text {CCS}}(\Gamma )$ be such that $K_N \subseteq K_{N+1}$ for all $N \in {\mathbb {N}}$ and $\Gamma = \bigcup _{N} K_N$. Lemma 5.2 yields that ${\mathcal {W}} = (e^{h_{-K_N}})_{N \in {\mathbb {N}}}$ is an increasing weight system. We set $C_\Gamma ({\mathbb {R}}^d) \mathrel {\mathop :}={\mathcal {W}}C({\mathbb {R}}^d)$. Clearly, the definition of $C_\Gamma ({\mathbb {R}}^d)$ is independent of the chosen sequence $(K_N)_{N \in {\mathbb {N}}}$. The next result is the key observation of this article.

### Proposition 5.4

Let $\emptyset \ne \Gamma \subseteq {\mathbb {R}}^d$ be open and convex and let $\psi \in {\mathcal {D}}({\mathbb {R}}^d)$. Then, the mappings

$$\begin{aligned} V_\psi :{\mathcal {S}}'(\Gamma ) \rightarrow C_\Gamma ({\mathbb {R}}^d_x) {\widehat{\otimes }}_\varepsilon {\mathcal {V}}_{{\text {pol}}}C({\mathbb {R}}^d_\xi ) \end{aligned}$$

and

$$\begin{aligned} V^*_\psi :C_\Gamma ({\mathbb {R}}^d_x) {\widehat{\otimes }}_\varepsilon {\mathcal {V}}_{{\text {pol}}}C({\mathbb {R}}^d_\xi ) \rightarrow {\mathcal {S}}'(\Gamma ) \end{aligned}$$

are well-defined and continuous.

We need some preparation for the proof of Proposition 5.4. Firstly, Lemma 2.1 implies that the topology of $C_\Gamma ({\mathbb {R}}^d_x) {\widehat{\otimes }}_\varepsilon {\mathcal {V}}_{{\text {pol}}}C({\mathbb {R}}^d_\xi )$ is generated by the system of seminorms

$$\begin{aligned} \Vert f \Vert _{K,v} \mathrel {\mathop :}=\sup _{(x,\xi ) \in {\mathbb {R}}^{2d}} \left| f(x,\xi )\right| e^{h_{-K}(x)} v(\xi ) < \infty , \qquad K \in CCS(\Gamma ), v \in {\overline{V}}({\mathcal {V}}_{{\text {pol}}}). \end{aligned}$$

For $k,n \in {\mathbb {N}}$ we write

$$\begin{aligned} \Vert \varphi \Vert _{{\mathcal {S}}^n_k} \mathrel {\mathop :}=\max _{\left| \alpha \right| \le n} \sup _{x \in {\mathbb {R}}^d} \left| \partial ^\alpha \varphi (x)\right| (1+\left| x\right| )^k, \qquad \varphi \in {\mathcal {S}}({\mathbb {R}}^d). \end{aligned}$$

The topology of ${\mathcal {S}}({\mathbb {R}}^d)$ is generated by the system of seminorms $\{ \Vert \,\cdot \, \Vert _{{\mathcal {S}}^n_k} \, | \, k,n \in {\mathbb {N}}\}$. We now give two technical lemmas.

### Lemma 5.5

Let $\psi \in {\mathcal {D}}({\mathbb {R}}^d)$, $K \subset {\mathbb {R}}^d$ be compact, $v \in {\overline{V}}({\mathcal {V}}_{{\text {pol}}})$ and $\varepsilon > 0$. Then,

$$\begin{aligned} \{ e^{\eta \cdot (t-x)} \overline{M_\xi T_x\psi }(t) e^{-\varepsilon \left| x\right| }v(\xi ) \, | \, (x,\xi ) \in {\mathbb {R}}^{2d}, \eta \in K\} \in {\mathfrak {B}}({\mathcal {S}}({\mathbb {R}}^d_t)). \end{aligned}$$

### Proof

Choose $r > 0$ such that ${\text {supp}} \psi \subseteq {\overline{B}}(0,r)$ and $R \ge 1$ such that $K \subseteq {\overline{B}}(0,R)$. For all $k,n \in {\mathbb {N}}$ we have that

$$\begin{aligned}&\sup _{(x,\xi ) \in {\mathbb {R}}^{2d}} \sup _{\eta \in K}e^{-\varepsilon \left| x\right| }v(\xi ) \Vert e^{\eta \cdot (t-x)} \overline{M_\xi T_x\psi }(t) \Vert _{{\mathcal {S}}^n_{k,t}} \le \sup _{(x,\xi ) \in {\mathbb {R}}^{2d}} \sup _{\eta \in K}e^{-\varepsilon \left| x\right| }v(\xi ) \cdot \\&\max _{\left| \alpha \right| \le n} \sup _{x \in {\mathbb {R}}^d} \sum _{\beta \le \alpha } \sum _{\gamma \le \beta } \left( {\begin{array}{c}\alpha \\ \beta \end{array}}\right) \left( {\begin{array}{c}\beta \\ \gamma \end{array}}\right) \left| \eta \right| ^{\left| \alpha \right| -\left| \beta \right| } e^{\eta \cdot (t-x)} (2\pi \left| \xi \right| )^{\left| \gamma \right| } \left| \partial ^{\beta -\gamma } {\overline{\psi }}(t-x)\right| (1+\left| t\right| )^k \\&\le e^{Rr} (8\pi R)^n \max _{\left| \alpha \right| \le n } \Vert \partial ^\alpha {\overline{\psi }} \Vert _{L^\infty } (1+r)^k \sup _{x \in {\mathbb {R}}^d} e^{-\varepsilon \left| x\right| }(1+\left| x\right| )^k \sup _{\xi \in {\mathbb {R}}^d}v(\xi ) (1+\left| \xi \right| )^n \\&< \infty . \end{aligned}$$

$$\square $$

### Lemma 5.6

Let $\psi \in {\mathcal {D}}({\mathbb {R}}^d)$ and $\eta \in {\mathbb {R}}^d$. Then, for all $k,n \in {\mathbb {N}}$ and $\varphi \in {\mathcal {S}}({\mathbb {R}}^d)$,

$$\begin{aligned} \left| V_{{\overline{\psi }},t}( e^{-\eta \cdot t} \varphi (t))(x, -\xi )\right| \le \frac{C_{\eta ,k,n,\psi }e^{-\eta \cdot x} \Vert \varphi \Vert _{{\mathcal {S}}^n_k}}{(1+\left| x\right| )^k(1+\left| \xi \right| )^n}, \qquad (x,\xi ) \in {\mathbb {R}}^{2d}, \end{aligned}$$

where

$$\begin{aligned} C_{\eta ,k,n,\psi } = 4^n(1+\sqrt{d})^n \max \{1,\left| \eta \right| ^n\} \max _{\left| \alpha \right| \le n} \Vert \partial ^\alpha \psi \Vert _{L^\infty } \int _{{\text {supp}} \psi } e^{-\eta \cdot t} (1+\left| t\right| )^k \mathrm{d}t . \end{aligned}$$

In particular, $\sup _{\eta \in K} C_{\eta ,k,n,\psi } < \infty$ for all $K \subset {\mathbb {R}}^d$ compact.

### Proof

We have that

$$\begin{aligned}&\left| V_{{\overline{\psi }},t}( e^{-\eta \cdot t} \varphi (t))(x, -\xi )\right| (1+\left| x\right| )^k (1 + \left| \xi \right| )^n \\&\le (1+ \sqrt{d})^n \max _{\left| \alpha \right| \le n}\left| \xi ^\alpha V_{{\overline{\psi }},t}( e^{-\eta \cdot t} \varphi (t))(x, -\xi )\right| (1+\left| x\right| )^k \\&\le (1+ \sqrt{d})^n (1+\left| x\right| )^k\max _{\left| \alpha \right| \le n} \sum _{\beta \le \alpha } \sum _{\gamma \le \beta } \left( {\begin{array}{c}\alpha \\ \beta \end{array}}\right) \left( {\begin{array}{c}\beta \\ \gamma \end{array}}\right) \cdot \\&\int _{{\mathbb {R}}^d} \left| \eta \right| ^{\left| \gamma \right| }e^{-\eta \cdot t} \left| \partial ^{\beta -\gamma }\varphi (t)\right| \left| \partial ^{\alpha -\beta }\psi (t-x)\right| \mathrm{d}t \\&\le (1+ \sqrt{d})^n (1+\left| x\right| )^k\max _{\left| \alpha \right| \le n} \sum _{\beta \le \alpha } \sum _{\gamma \le \beta } \left( {\begin{array}{c}\alpha \\ \beta \end{array}}\right) \left( {\begin{array}{c}\beta \\ \gamma \end{array}}\right) \cdot \\&\int _{{\text {supp}} \psi } \left| \eta \right| ^{\left| \gamma \right| }e^{-\eta \cdot (t+x)} \left| \partial ^{\beta -\gamma }\varphi (t+x)\right| \left| \partial ^{\alpha -\beta }\psi (t)\right| \mathrm{d}t \\&\le C_{\eta ,k,n,\psi }e^{-\eta \cdot x} \Vert \varphi \Vert _{{\mathcal {S}}^n_k}. \end{aligned}$$

$$\square $$

### Proof of Proposition 5.4

(i) $V_\psi :{\mathcal {S}}'(\Gamma ) \rightarrow C_\Gamma ({\mathbb {R}}^d_x) {\widehat{\otimes }}_\varepsilon {\mathcal {V}}_{{\text {pol}}}C({\mathbb {R}}^d_\xi )$ *is well-defined and continuous*: Let $K \in {\text {CCS}}(\Gamma )$ and $v \in {\overline{V}}({\mathcal {V}}_{{\text {pol}}})$ be arbitrary. Choose $\varepsilon > 0$ so small that $K_\varepsilon \in {\text {CCS}}(\Gamma )$ and pick, for $x \in {\mathbb {R}}^d$ fixed, $\eta _x \in K$ such that $h_{-K}(x) \le (-\eta _x \cdot x) + 1$. Example 5.3 implies that, for all $f \in {\mathcal {S}}'(\Gamma )$ and $(x,\xi ) \in {\mathbb {R}}^{2d}$,

$$\begin{aligned} \left| V_\psi f(x,\xi )\right| e^{h_{-K}(x)}v(\xi )&= \left| \langle e^{-(\eta _x - \varepsilon \frac{x}{\left| x\right| }) \cdot t} f(t), e^{(\eta _x - \varepsilon \frac{x}{\left| x\right| }) \cdot t} \overline{M_\xi T_x\psi }(t) \rangle \right| e^{h_{-K}(x)}v(\xi ) \\&\le e \left| \langle e^{-(\eta _x - \varepsilon \frac{x}{\left| x\right| }) \cdot t} f(t), e^{(\eta _x - \varepsilon \frac{x}{\left| x\right| }) \cdot (t-x)} \overline{M_\xi T_x\psi }(t) \rangle \right| e^{-\varepsilon \left| x\right| } v(\xi ) \\&\le ep_{K_\varepsilon ,B}(f), \end{aligned}$$

where

$$\begin{aligned} B = \{ e^{\tau \cdot (t-x)} \overline{M_\xi T_x\psi }(t) e^{-\varepsilon \left| x\right| }v(\xi ) \, | \, (x,\xi ) \in {\mathbb {R}}^{2d}, \tau \in K_\varepsilon \} \in {\mathfrak {B}}({\mathcal {S}}({\mathbb {R}}^d_t)) \end{aligned}$$

by Lemma 5.5.

(ii) $V^*_\psi :C_\Gamma ({\mathbb {R}}^d_x) {\widehat{\otimes }}_\varepsilon {\mathcal {V}}_{{\text {pol}}}C({\mathbb {R}}^d_\xi ) \rightarrow {\mathcal {S}}'(\Gamma )$ *is well-defined and continuous*: We start by showing that $V^*_\psi F \in {\mathcal {S}}'(\Gamma )$ for all $F \in C_\Gamma ({\mathbb {R}}_x^d) {\widehat{\otimes }}_\varepsilon {\mathcal {V}}_{{\text {pol}}}C({\mathbb {R}}_\xi ^d)$. Lemma 5.6 implies that, for all $\eta \in \Gamma$,

$$\begin{aligned} \langle f_\eta , \varphi \rangle = \int \int _{{\mathbb {R}}^{2d}} F(x,\xi ) V_{{\overline{\psi }},t}( e^{-\eta \cdot t} \varphi (t))(x, -\xi ) \,\mathrm{d}x \,\mathrm{d}\xi , \qquad \varphi \in {\mathcal {S}}({\mathbb {R}}^d), \end{aligned}$$

is a well-defined continous linear functional on ${\mathcal {S}}({\mathbb {R}}^d)$. Since $e^{-\eta \cdot t}V^*_\psi F(t) = {f_\eta (t)}|_{{\mathcal {D}}({\mathbb {R}}^d)}$, we obtain that $e^{-\eta \cdot t}V^*_\psi F(t) \in {\mathcal {S}}'({\mathbb {R}}^d)$ and that

$$\begin{aligned} \langle e^{-\eta \cdot t}V^*_\psi F(t), \varphi (t) \rangle = \int \int _{{\mathbb {R}}^{2d}} F(x,\xi ) V_{{\overline{\psi }},t}( e^{-\eta \cdot t} \varphi (t))(x, -\xi ) \,\mathrm{d}x \,\mathrm{d}\xi , \qquad \varphi \in {\mathcal {S}}({\mathbb {R}}^d). \end{aligned}$$

Next, we show that $V^*_\psi$ is continuous. Let $K \in {\text {CCS}}(\Gamma )$ and $B \in {\mathfrak {B}}({\mathcal {S}}({\mathbb {R}}^d))$ be arbitrary. Choose $\varepsilon > 0$ so small that $K_\varepsilon \in {\text {CCS}}(\Gamma )$. Lemma 5.6 implies that there is $v \in {\overline{V}}({\mathcal {V}}_{{\text {pol}}})$ such that

$$\begin{aligned} \left| V_{{\overline{\psi }}}(e^{-\eta \cdot t} \varphi (t))(x, -\xi )\right| \le e^{h_{-K}(x)} v(\xi ), \qquad (x,\xi ) \in {\mathbb {R}}^{2d}, \end{aligned}$$

for all $\eta \in K$ and $\varphi \in B$. Set $w(\xi ) = v(\xi ) (1+ \left| \xi \right| )^{d+1} \in {\overline{V}}({\mathcal {V}}_{{\text {pol}}})$. Example 5.3 implies that, for all $F \in C_\Gamma ({\mathbb {R}}_x^d) {\widehat{\otimes }}_\varepsilon {\mathcal {V}}_{{\text {pol}}}C({\mathbb {R}}_\xi ^d)$,

$$\begin{aligned} p_{K,B}(V^*_\psi F)&\le \sup _{\eta \in K} \sup _{\varphi \in B} \int \int _{{\mathbb {R}}^{2d}} \left| F(x,\xi )\right| \left| V_{{\overline{\psi }},t}( e^{-\eta \cdot t} \varphi (t))(x, -\xi )\right| \,\mathrm{d}x \,\mathrm{d}\xi \\&\le \int \int _{{\mathbb {R}}^{2d}} \left| F(x,\xi )\right| e^{h_{-K}(x)} v(\xi ) \,\mathrm{d}x \,\mathrm{d}\xi \le C \Vert F \Vert _{K_\varepsilon , w}, \end{aligned}$$

where

$$\begin{aligned} C = \int _{{\mathbb {R}}^d} e^{-\varepsilon \left| x\right| } dx \int _{{\mathbb {R}}^d} \frac{1}{(1+\left| \xi \right| )^{d+1}} \mathrm{d}\xi . \end{aligned}$$

$$\square $$

We now combine Theorem 4.1 with the results from Sect. 4 to study the space ${\mathcal {S}}'(\Gamma )$. Let $\emptyset \ne \Gamma \subseteq {\mathbb {R}}^d$ be open and convex and let $(K_N)_{N \in {\mathbb {N}}} \subset {\text {CCS}}(\Gamma )$ be such that $K_N \subseteq K_{N+1}$ for all $N \in {\mathbb {N}}$ and $\Gamma = \bigcup _{N} K_N$. For ${\mathcal {W}} = (e^{h_{-K_N}})_{N \in {\mathbb {N}}}$ we set ${\mathcal {B}}'_\Gamma ({\mathbb {R}}^d) \mathrel {\mathop :}={\mathcal {B}}'_{{\mathcal {W}}}({\mathbb {R}}^d)$ and ${\mathcal {O}}'_{C, \Gamma }({\mathbb {R}}^d) = {\mathcal {O}}'_{C, {\mathcal {W}}}({\mathbb {R}}^d)$. Clearly, these definitions are independent of the chosen sequence $(K_N)_{N \in {\mathbb {N}}}$. We are ready to state and prove our main theorem.

### Theorem 5.7

Let $\emptyset \ne \Gamma \subseteq {\mathbb {R}}^d$ be open and convex. Then, ${\mathcal {S}}'(\Gamma ) = {\mathcal {B}}'_\Gamma ({\mathbb {R}}^d) = {\mathcal {O}}'_{C, \Gamma }({\mathbb {R}}^d)$ as locally convex spaces and ${\mathcal {S}}'(\Gamma )$ is an ultrabornological (PLS)-space.

### Proof

Let $(K_N)_{N \in {\mathbb {N}}} \subset {\text {CCS}}(\Gamma )$ be such that $K_N \subseteq K_{N+1}$ for all $N \in {\mathbb {N}}$ and $\Gamma = \bigcup _{N} K_N$. Set ${\mathcal {W}} = (e^{h_{-K_N}})_{N \in {\mathbb {N}}}$. Lemma 5.2 and Example 5.3 imply that ${\mathcal {W}}$ satisfies (2.1), (2.2) and (2.3). Hence, in view of the reconstruction formula (3.1), the topological identity ${\mathcal {S}}'(\Gamma ) = {\mathcal {O}}'_{C, \Gamma }({\mathbb {R}}^d)$ follows from Proposition 4.1 and Proposition 5.4. Since ${\mathcal {W}}$ also satisfies (4.1) (again by Lemma 5.2 and Example 5.3), the other statements are a direct consequence of Theorem 4.2. $\square$

## Footnotes

1 To be precise, the spaces considered in [9], denoted there by $(\dot{{\mathcal {B}}}_{{\mathcal {W}}^\circ } ({\mathbb {R}}^d))'$ and ${\mathcal {O}}'_C({\mathcal {D}}, L^1_{{\mathcal {W}}})$, differ from ${\mathcal {B}}'_{{\mathcal {W}}}({\mathbb {R}}^d)$ and ${\mathcal {O}}'_{C,{\mathcal {W}}}({\mathbb {R}}^d)$ defined above. However, if ${\mathcal {W}}$ satisfies (2.1), (2.2) and (2.3), then ${\mathcal {B}}'_{{\mathcal {W}}}({\mathbb {R}}^d) = (\dot{{\mathcal {B}}}_{{\mathcal {W}}^\circ } ({\mathbb {R}}^d))'$ and ${\mathcal {O}}'_C({\mathcal {D}}, L^1_{{\mathcal {W}}}) = {\mathcal {O}}'_{C,{\mathcal {W}}}({\mathbb {R}}^d)$; the first equality is clear, while the second one follows from [9, Prop. 6.2]. Moreover, under these conditions, all statements and proofs from [9] remain valid if one replaces $L^1_{{\mathcal {W}}}({\mathbb {R}}^d)$ by ${\mathcal {W}}C({\mathbb {R}}^d)$.
